# Generation of an engineered food-grade *Lactococcus lactis* strain for production of an antimicrobial peptide: in vitro and in silico evaluation

**DOI:** 10.1186/s12896-020-00612-3

**Published:** 2020-03-30

**Authors:** Abbas Tanhaeian, Mehdi Mirzaii, Zana Pirkhezranian, Mohammad Hadi Sekhavati

**Affiliations:** 1 Department of Biotechnology and Plant Breeding, Faculty of Agriculture, Ferdowsi University of Mashhad, P.O. Box 91775-1163, Mashhad, Iran; 2 School of Medicine, Shahroud University of Medical Sciences, Shahroud, Iran; 3 Department of Animal Sciences, Faculty of Agriculture, Ferdowsi University of Mashhad, P.O. Box 91775-1163, Mashhad, Iran

**Keywords:** Food-grade *L. lactis*, Antimicrobial peptide, Biofilm formation, Antioxidant activity, MD simulation

## Abstract

**Background:**

Foodborne pathogens and their biofilms are considered as one of the most serious problems in human health and food industry. Moreover, safety of foods is a main global concern because of the increasing use of chemical food additives. Ensuring food safety enhances interest in discovery of new alternative compounds such as antimicrobial peptides (AMPs), which can be used as bio-preservatives in the food industry. In this study, the most important antimicrobial peptides of camel milk lactoferrin (lactoferrampin and lactoferricin) were recombinantly expressed in the form of chimeric peptide (cLFchimera) in a food-grade *L. lactis* strain. P170 expression system was used to express secreted cLFchimera using pAMJ1653 expression vector which harbors a safe (non-antibiotic) selectable marker.

**Results:**

Peptide purification was carried out using Ni-NTA agarose column from culture medium with concentration of 0.13 mg/mL. The results of disk diffusion test revealed that cLFchimera had considerable antimicrobial activity against a number of major foodborne bacteria. Furthermore, this chimeric peptide showed strong and weak inhibitory effect on biofilm formation against *P. aeruginosa*, *S. aureus E. faecalis,* and *E. coli,* respectively. Antioxidant activity and thermal stability of the chimeric peptide was determined. The results showed that cLFchimera had antioxidant activity (IC_50_: 310 μ/mL) and its activity was not affected after 40 min of boiling. Finally, we evaluated the interaction of the peptide with LPS and DNA in bacteria using molecular dynamic simulation as two main intra and extra cellular targets for AMPs, respectively. Our in silico analysis showed that cLFchimera had strong affinity to both of these targets by positive charged residues after 50 ns molecular dynamic simulation.

**Conclusions:**

Overall, the engineered food-grade *L. lactis* generated in the present study successfully expressed a secreted chimeric peptide with antimicrobial properties and could be considered as a promising bio-preservative in the food industry.

## Background

Foodborne pathogens and their biofilms are one of the serious problems of human health. Due to production of bacterial matrix during biofilm formation, bacteria are well protected against clinical antibiotics and thus it is difficult to eliminate them from food processing facilities [43], so introduction of effective methods is critical to prevent and remove biofilms to guarantee safe food production and preservation process [24, 44].

Today, shelf life and the safety of food products were enhanced by using natural or controlled microflora, mostly lactic acid bacteria [47] and natural components such as antimicrobial peptides (AMPs) [16].

As *Lactococcus lactis* are normally present in dairy products, replacement and establishment of these bacteria in the intestine can help human health by preventing invasion of pathogenic bacteria using reduction of intestine pH as well as production of natural antimicrobial compounds [7]. AMPs are a group of innate immune system molecules that contain 12–50 amino acids and exist in all organisms [38]. AMP has been known as a natural molecule with activity toward a broad spectrum of microorganisms including bacteria, fungi, and viruses [11]. Moreover, AMPs have been considered as a new generation of biologically active regulators that can prevent oxidation and microbial degradation in foods [35, 49].

In the present study, the recombinant expression of a chimeric form of peptide derived from camel milk lactoferrin (lactoferrampin + lactoferricin named cLFchimera) was achieved in a food-grade *L. lactis* strain. Lactoferrin has the ability to modulate the immune system as well as bacteriostatic activity [41, 53]. Lactoferrampin and lactoferricin are two rich sources of hydrophobic and cationic antimicrobial peptides in N-terminus lactoferrin protein with activity toward a broad-spectrum of microorganisms including bacteria, fungi and viruses [8, 69]. Haney and co-workers [22] fused these two peptides and showed that the chimeric form has stronger antimicrobial activity compared to natural lactoferrin [22].

More recently, the recombinant form of cLFchimera has been cloned and expressed in *E. coli* [64] in our lab. The results of in vitro studies showed that this peptide has antibacterial [62, 64, 65], antiviral [61], and anticancer [63] properties. Furthermore, the results of an in vivo experiment showed that supplementing *E. coli* challenged broilers with cLFchimera improved villi morphology in the jejunum, restored microbial balance in the ileum, and improved gene expression of cytokines and tight junctions in the jejunum of challenged birds [14].

In this investigation, we generated an engineered food-grade *L. lactis* with the ability to secrete a chimeric peptide derived from camel lactoferrin into the culture medium. in vitro antibacterial, anti-biofilm and antioxidant activity of the recombinant chimeric peptide were determined on some food spoilage bacterial strains. Finally, using computational modeling approaches we try to predict peptide interaction to lipopolysaccharides (LPS) and DNA as two main targets in bacteria [15, 36, 50, 51].

## Methods

### Bacterial strains, vectors, growth conditions and other reagents

**Fig. 1:**
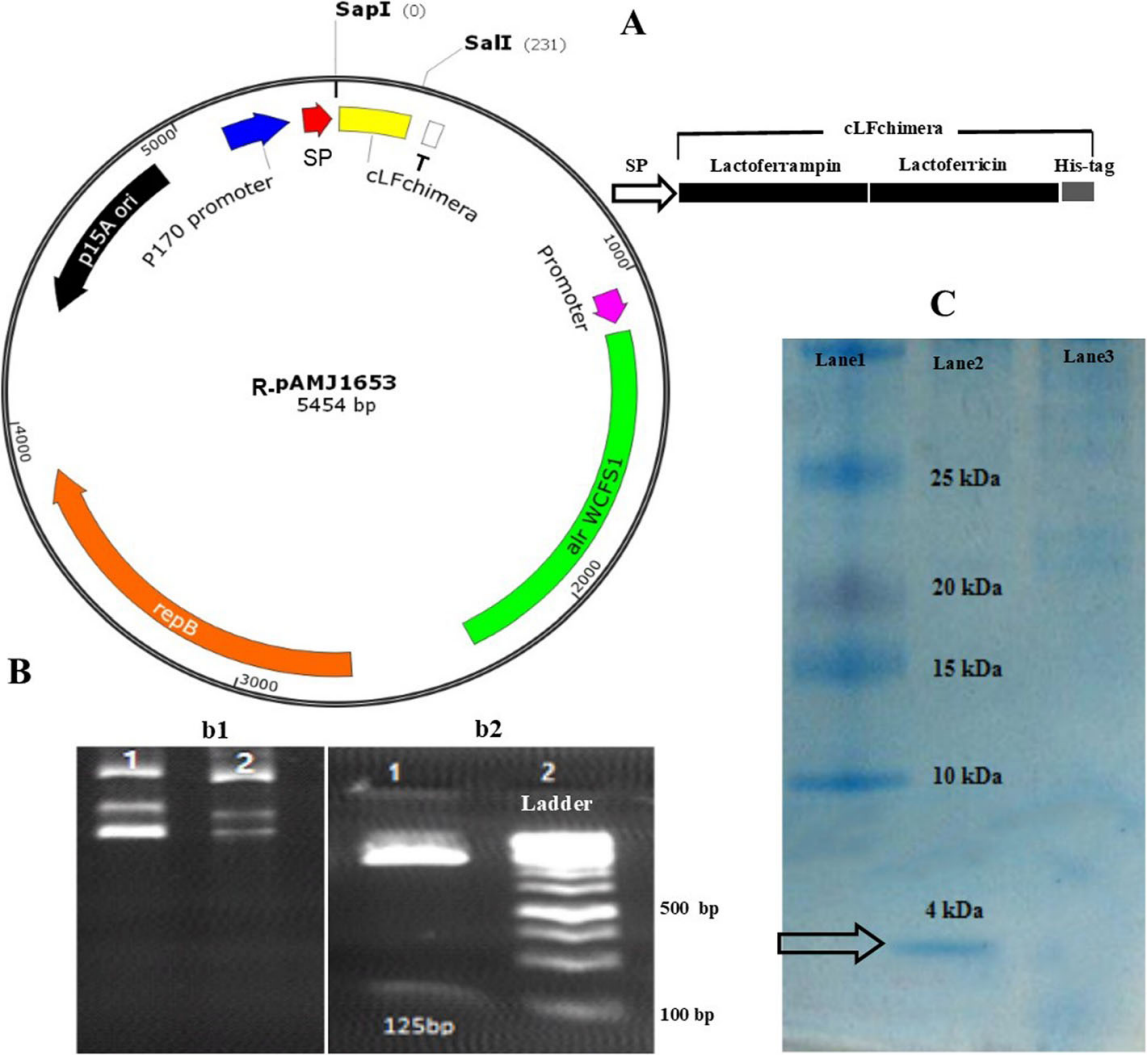
**A** The schematic maps of recombinant pAMJ1653 vector (R-pAMJ1653) harboring cLFchimera and schematic representation of cLFchimera and its parts. P170 promoter, *L. lactis* promoter is up-regulated by low-pH; SP, signal sequence of SP310mut2; LFchimera, chimeric antimicrobial peptide, T, terminator; alr WCFS1, *L. plantarum* WCFS1 *alr* gene encoding alanine racemase; repB, a replicon from *L. lactis* for maintenance in *L. lactis;* p15A, a replicon from *E. coli* for maintenance in *E. coli.***B** Restriction mapping analysis. **b1**) Undigested pAMJ1653 plasmid (Lane 1 and 2, refer to replicates); **b2**) Double digestion of recombinant pAMJ1653 vector by *SapI* and *SalI*. The size of the band of interest after double digestion is 125 bp. **C** SDS-PAGE analysis of the *L. lactis* culture supernatant. A protein band with the size of 4.2 kDa (shown by arrow) represents the recombinant cLFchimera peptide. From left to right: lane 1: size marker protein (ladder protein), lane 2: purified cLFchimera from culture supernatant of *L. lactis* harboring recombinant pAMJ1653 vector and lane 3: culture supernatant of *L. lactis* harboring self-ligated pAMJ1653 vector with no cLFchimera coding sequence was passed through Ni-NTA agarose column and was loaded on SDS-PAGE as negative control

*Lactococcus lactis* AMJ1543 (Bioneer, Denmark) strain was used as the expression host. *L. lactis* strain AMJ1543 was grown at 30 °C in rich M17 medium supplemented with glucose and D-Alanine (2.25 mM, Sigma, USA). This strain is a D-Alanine auxotrophic strain and possesses a non-antibiotic-based alanine racemase selection system. The *alr* gene encodes alanine racemase protein which catalyzes the interconversion of L-Alanine to D-Alanine which is crucial for cell wall biosynthesis. D-Alanine is not a common ingredient in large-scale fermentation media, so the *L. lactis* strain AMJ1543 is not able to grow in the medium free of D-Alanine. Presence of pAMJ1653 expression vector which harbors *alr* encoding gene in *L. lactis* strain AMJ1543 can provide a condition in which L-Alanine can be converted to D-Alanine by alanine racemase protein, so that it can be grown in M17 (Sigma, USA) medium without D-Alanine. The pAMJ1653 vector (Bioneer, Denmark, Fig. 1A) was used as an expression vector which contains specific *L. lactis* promoter and is up-regulated by low-pH [29]. Unless indicated otherwise, all chemicals, commercial kits and enzymes were obtained from Sigma Chemical (USA), Roche (Germany), New England Biolabs (England) and Thermo Fisher Scientific (USA) Companies, respectively.

#### Gene synthesis and vector construction

The chimeric lactoferricin and lactoferrampin consists of 36 amino acids and was generated through the fusion of two short regions of camel lactoferrin ^284^DLIWKLLVKAQEKFGRGKPS^303^ (ID: AHJ37525) and ^49^RVKKMRRQWQACKSS^35^ (ID:NP_001290496.1) linked by Lysine (GenBank accession number: MH327768). The chimeric peptide encoding sequence was codon optimized for the appropriate expression in *L. lactis* by Genscript® (USA). The restriction sites of *Sap*I and *Sal*I were added to the N and C-terminus of the chimera sequence for cloning in pAMJ1653 vector. The sequence was chemically synthesized by Generay Biotech (Shanghai, China). For vector construction, pAMJ1653 and pGH cloning vectors (a vector harboring synthetic gene) were sequentially digested by *Sap*I (New England Biolabs, England) and *Sal*I restriction enzymes (Thermo Fisher Scientific, USA). The digestion products were then purified and ligated by gel extraction and fast ligation kits (Thermo Fisher Scientific, USA), respectively.

### Peptide expression

The competent cells of *L. lactis* strain AMJ1543 were initially prepared [27] and subsequently transformed by electroporation with an aliquot of the ligation reaction based on the protocol of electroporation for *L. lactis* strains [39]. The colonies harboring recombinant pAMJ1653 were first selected in growth medium free of D-alanine (M17 + glucose) at 30 °C and then verified by colony polymerase chain reaction (PCR). The sequences of primers for recombinant constructs verification were PLF: 5′-CTGCCTCCTCTCCCTAGTGC-3′ for the forward and PLR: 5′-CTAAGGATGATTTCTGGCAGGG-3′ for the reverse primer, respectively. PCR program was performed using the Personal Cycler™ thermo cycler (Biometra, Germany) with initial denaturation at 94 °C for 5 min followed by 35 cycles of denaturation, annealing and extension for 30 s at 94 °C, 30 s at 56 °C and 30 s at 72 °C, respectively. Finally, an additional cycle extension was carried out for 10 min at 72 °C. The total volume of PCR reaction was 25 μL with the reaction mixture containing 2.5 μL of 10X PCR buffer, 2 μL MgCl_2_ (50 mM), 2 μL dNTPs (2.5 pmol/μL), 1.5 μL of mix primer (5 pmol/μL), 0.125 U/μL of EX Taq DNA polymerase (Takara, Japan) and deionized water up to 25 μL reaction volume. The culture supernatant obtained from the transformant strain of *L. lactis* was first evaluated for the production and accumulation of the heterologous peptide using the disk diffusion test, and subsequently analyzed by SDS polyacrylamide gel electrophoresis (SDS-PAGE). The recombinant *L. Lactis* was grown in M17 medium at 30 °C for 16 h and then 15 μL of culture supernatant was electrophoresed on SDS-PAGE in Tris/glycine/SDS buffer using 17.5% acrylamide gels and visualized using Coomassie Brilliant Blue staining protocol. The expressed peptide was purified using Ni-NTA agarose column (Thermo, USA) according to the manufacturer’s instructions. The quality and quantity of purified recombinant cLFchimera was analyzed on a 17.5% SDS-PAGE gel electrophoresis and Bradford method [9], respectively.

### Antibacterial activity tests

The foodborne bacterial strains: *Escherichia coli* (ATCC 25404), *Staphylococcus aureus* (ATCC 15981), *Salmonella typhimurium* (ATCC 14028), *Enterococcus faecalis* (ATCC 47077)*, Listeria monocytogenes* (ATCC 19111) and *Pseudomonas aeroginosa* PAO1 were kindly provided from the bacterial collection in the Department of Food Science and Technology, Ferdowsi University of Mashhad, Iran. The disc diffusion method was performed to determine the rate of bacterial inhibition by culture supernatant. Various volumes of culture supernatant of transformed cells (10, 20 and 40 μL) were loaded as peptide solution on 6 mm sterile paper discs. Each loaded disc was placed on the surface of a Muller Hinton Agar petri dish and incubated at 37 °C for 16 h. The discs loaded by standard antibiotic Penicillin (10 mg/disc) and Gentamicin (10 mg/disc) as well as 40 μL of culture supernatant obtained from transformed *L. lactis* by vector with no cLFchimera coding sequence were used as positive and negative control respectively. Antimicrobial activity was evaluated by measuring the diameter of the inhibition zone around the discs (mm). As maintained by the Clinical and Laboratory Standards Institute (CLSI) the minimum inhibitory concentrations (MICs) of the cLFchimera were measured in Broth microdilution [17, 28]. In summary, Mueller Hinton Broth (Cation-adjusted) that contains increasing concentrations of cLFchimera is inoculated with specific number of cells (approx. 5 × 10^5^ CFUs/mL) in micro-titer plates (polypropylene), while each plate includes a positive and negative control. After incubation, the MIC concentration of AMPs is defined as the lowest concentration inhibiting visible growth of bacteria after overnight incubation. All plates were incubated for 18–20 h. The MIC measurements were carried out in triplicate.

### Phenotypic biofilm assay

Semi-quantitative determination of biofilm formation was performed in triplicate by colorimetric microtiter plate as previously described [30]. Pure cultures of *S. aureus* ATCC 15981, *E. coli* ATCC 25404, *P. aeroginosa* PAO1 and *E. faecalis* ATCC 47077 were used. Briefly, the strains were cultured in 5 mL tryptic soy broth (TSB; DIFCO, Becton Dickinson, Franklin Lakes, NJ, USA) with 1% glucose (Glc) for 18 h at 37 °C and treated with cLFchimera (50% of the MIC) or control medium. The cultures were diluted (1:100) in the same medium, 200 μL was inoculated in a 96-well plate, and plates were incubated at 37 °C for 24 h. The plates were washed twice with phosphate-buffered saline (PBS), dried for 1 h at 65 °C, 1% crystal violet was added, and the plates were incubated for a further 30 min at 25 °C. Each well was washed twice with PBS and 200 μL PBS was added prior to measuring absorbance at 590 nm using a microplate reader (state fax3100, USA). All strains were tested in quadruplicate in two independent experiments. The cut-offs proposed by Stepanović et al. [60] were used to classify the level of biofilm production. The uninoculated medium was used, as control, to determine the background OD. The cut-off OD (ODc) was defined as three standard deviations above the mean OD of the negative control and the final OD value, of a tested strain, was defined as the average OD of the strain reduced by the ODc value. The adherence ability of the tested strain was classified into four categories based on the OD: non-adherent (OD < ODc), weakly adherent (ODc < OD <2XODc), moderately adherent (2XODc < OD < 4XODc), and strongly adherent (4XODc < OD).

### Antioxidant activity of cLFchimera

The antioxidant activity was determined using 2, 2-Diphenyl-l-picrylhydrazyl (DPPH) as a free radical as previously described [10]. Briefly, peptide solution in methanol (0.1 mL) was added to 3.9 mL of a 6 × 10^− 5^ mol/L methanol DPPH solution. The decrease in absorbance was determined at 515 nm at 0 min, 1 min and every 15 min until the reaction reached a plateau. Inhibitory effect of cLFchimera was determined as follows: [OD DPPH solution (control) – OD treatment (cLFchimera)/DPPH solution] 100%. Antiradical activity was defined as the amount of antioxidant necessary to decrease the initial DPPH concentration by 50% (Efficient Concentration = EC_50_).

### Thermal stability of recombinant peptide

Forty microliter volumes of the culture supernatant were boiled at 100 °C at different times including 0, 10, 20, and 40 min. The antibacterial activity was determined against *S. aureus* ATCC 25923 using disc diffusion method according to the method described above.

### In silico analysis

BDNA structure was obtained from the Protein Data Bank (1BNA). The cLFchimera were modeled using Modeller 9.2 [19]. LPS structure was derived from Gram-negative bacteria membrane which was obtained from Prof, Xalid Syma [33]. The accuracy of the predicted models was examined using Ramachandran plot analysis in PROCHECK http://servicesn.mbi.ucla.edu/PROCHECK/ [34]. The complex of BDNA-cLFchimera and LPS-CLFchimera was studied by molecular dynamics simulation (MD) with GROMACS 2016.1 package with periodic boundary conditions in all directions [1, 5, 70]. AMBER94 force field [21] for DNA*peptide and the GROMOS 53A6 force field [45] for LPS*peptide interactions were used. The long-range electrostatic interactions were calculated using particle-mesh Ewald (PMD) method, whereas the van der Waal interactions were treated with smooth cutoff at a distance of 12 A° [31]. All the systems were solvated in cubic water box with Simple Point Charge (SPC) water model [4]. To neutralize the entire system Na^+^ and Cl^−^ ions were added by substituting the water molecules. Energy minimization was performed using steepest descent algorithm for 50,000 cycles. Further, minimized system was equilibrated into the isothermal-isobaric (NPT) phases for 1000 ps at a constant pressure of 1 bar with Parrinello-Rahman pressure coupling method [42] and a temperature of 300 K with Nosé-Hoover temperature coupling method [20]. The equilibrated systems were used for a production run at 300 K and 1 bar pressure for 50,000 ps. To increase the accuracy every system was simulated in three replicates. Dynamic behavior and stability of each system were analyzed including root mean square deviation (RMSD), center of mass distances (COM) and hydrogen bond using Gromacs in-built tools. Binding free energy was calculated using molecular mechanics/Poisson Boltzmann surface area (MM/PBSA) estimation. The MmPbSaDecomp.py python script was used to estimate the contribution of each residue to the total binding free energy [31, 33].

### Statistical analysis

All assays were performed with three biological replications and the calculations were made to determine the average of diameters for inhibition zones. Data sets were subjected to analysis of variance (ANOVA) and Duncan’s multiple range test using SAS software (SAS 9.1).

## Results

### Design and vector construction

The results of restriction digestion (Fig. 1b2) and sequencing showed that cLFchimera coding sequence was successfully cloned into pAMJ1653 vector in the correct frame (Fig. 1A) without any mutation. The colonies transformed by pAMJ1653 vector were analyzed by colony PCR using P170 specific primers which amplified 125 bp fragment in culture medium without alanine.

### Transformation of *L. lactis* and peptide expression

*L. lactis* harboring recombinant pAMJ1653 vector was selected through colony PCR by specific primers on a single colony. The expression of the chimeric peptide in harvested supernatant was evaluated by SDS-PAGE analysis. As expected, a 4.2 kD protein band corresponding to the size of cLFchimera was observed in the gel; suggesting that the peptide was properly expressed in AMJ1543 strain and secreted into medium culture (Fig. 1C). The His-tag purified peptide concentration was 0.13 mg/mL.

### Evaluation of antibacterial activity

**Fig. 2:**
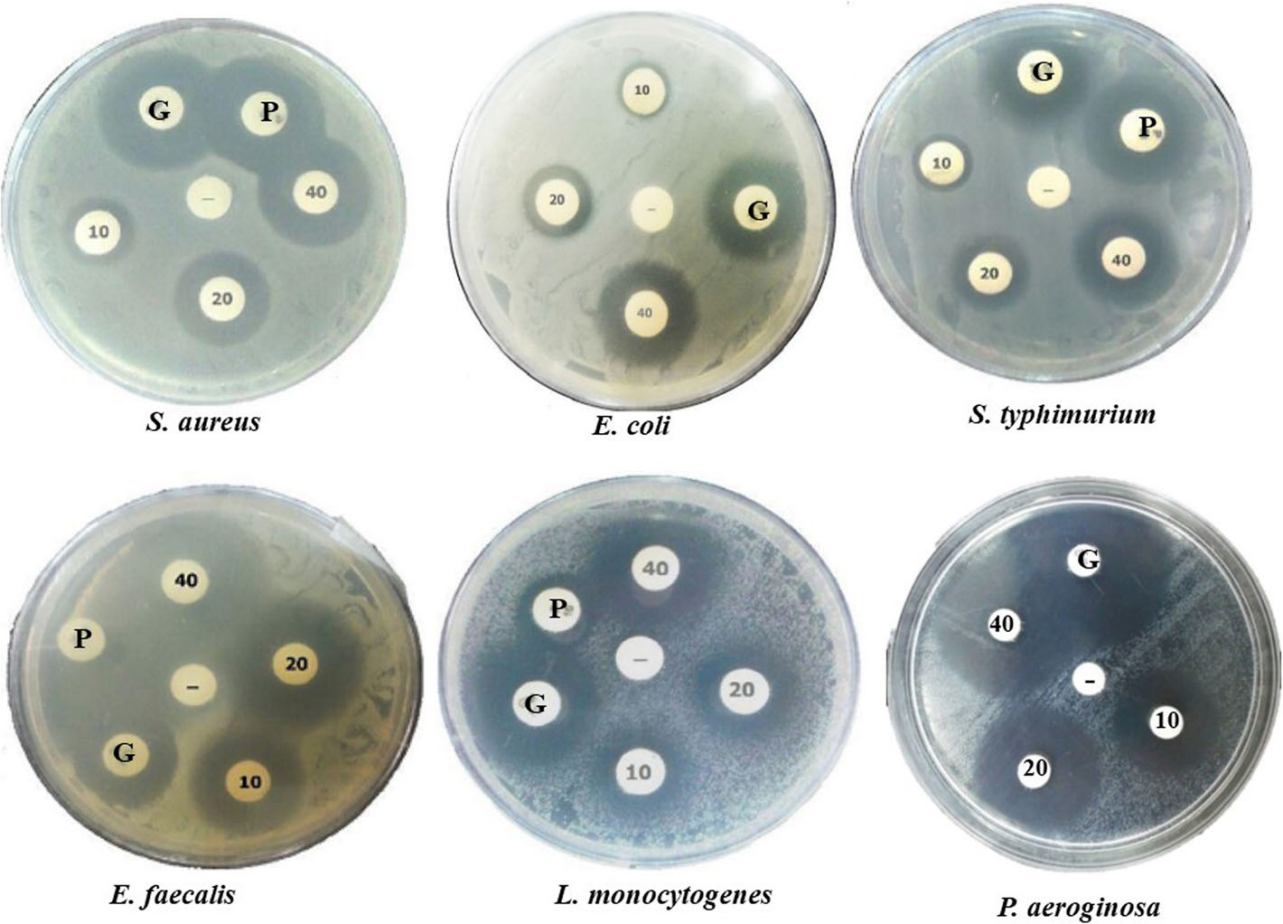
The result of disc diffusion assay for the candidate pathogens in this study. Three different volumes of culture supernatant containing the chimeric peptide (10, 20 and 40 μL) were considered as treatments. +C: Penicillin (P, 10 mg/disc) and Gentamicin (G, 10 mg/disc), −C: culture supernatant of *L. lactis* harboring self-ligated pAMJ1653 vector

For evaluation of peptide functionality, the medium containing cLFchimera was examined for antibacterial activity using disc diffusion assay. The results indicated that the medium containing the chimeric peptide had antibacterial effect against *E. coli* (ATCC 25404), *S. aureus* (ATCC 15981), *S. typhimurium* (ATCC 14028), *E. faecalis* (ATCC 47077), *L. monocytogenes* (ATCC 19111) and *P. aeroginosa PAO1* (Fig. 2).

Moreover, the statistical analysis of inhibition zone diameters showed a significant difference between diverse amounts of cultural medium containing recombinant peptide against all foodborne bacteria (*p* > 0.0001). The results are presented in the Table 1.

**Table 1:** Antimicrobial activity of the chimeric peptide evaluated by disc diffusion method

Bacterial species	Volume of supernatant (μL)			Positive control	Negative control
	10	20	40	Gentamicin	
*S. aureus*	11.1 ± 0.14 ^c^	15.8 ± 0.14 ^b^	21.1 ± 0.14^a^	20.1 ± 0.14 ^a^	_
*E. coli*	9.1 ± 0.17 ^c^	13.8 ± 0.17 ^b^	16.2 ± 0.17^a^	15.8 ± 0.17^a^	_
*S. typhimurium*	11.2 ± 0.33^c^	13.9 ± 0.33^b^	18.4 ± 0.33^a^	19.0 ± 0.33^a^	_
*L. monocytogenes*	11.1 ± 0.50 ^c^	16.6 ± 0.5 ^b^	21.3 ± 0.5 ^a^	21.3 ± 0.5 ^a^	_
*E. faecalis*	20.8 ± 0.32 ^d^	24.5 ± 0.32 ^c^	28.6 ± 0.32 ^b^	30.1 ± 0.32 ^a^	_
*P. aeroginosa*	21.0 ± 0.22^d^	24.2 ± 0.22^c^	27.6 ± 0.22 ^b^	31.1 ± 0.22 ^a^	_

The data are the inhibition zone around the discs (mm) loaded by different volume of supernatants containing recombinant peptide and antibiotic. The data are presented as average values of three replicates with their standard error. Negative control: M17 cultured with non-recombinant *L. lactis*. Means with different letter(s) are significantly different based on DUNCAN multiple test (α = 0.05). – representing no growth inhibition

### Phenotypic biofilm assay

**Fig. 3:**
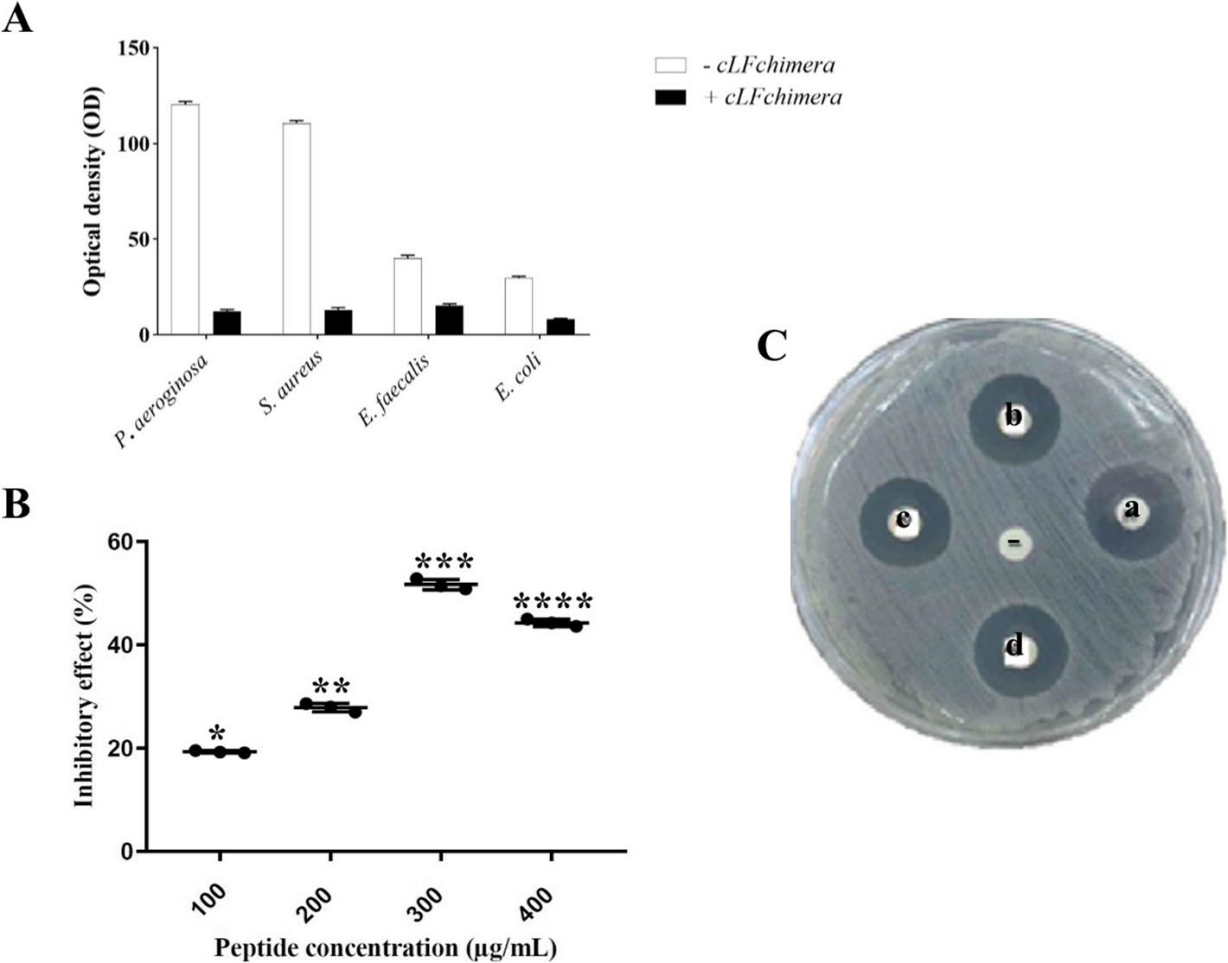
**A** Inhibitory effect of cLFchimera on biofilm formation which was determined by phenotypic biofilm assay. The absorbance was measured at 590 nm. +cLFchimera: treated with peptide; −cLFchimera: un-treated with peptide. **B** Inhibitory effect of cLFchimera on DPPH as a free radical. **C** The results of thermal stability of recombinant chimeric peptide against *S. aureus* for three different periods of boiling (a, b and c referred to 10, 20 and 40 min, respectively). d: Penicillin (10 mg/disc), −: culture supernatant of *L. lactis* harboring self-ligated pAMJ1653 vector. * It referred significantly to different levels of peptide concentration. The average percentage of inhibitory effect with different number of asterisk (s) is significantly different based on DUNCAN multiple test (α = 0.05)

The effect of cLFchimera on biofilm formation was evaluated against *P. aeruginosa, S. aureus*, *E. faecalis* and *E. coli* bacteria. To this end, MIC for cLFchimera was determined by purified cLFchimera (Table 2) and then the inhibitory effect of this peptide was examined on biofilm formation by phenotypic biofilm assay. Based on the phenotypic biofilm results, the highest and the lowest inhibitory effect of cLFchimera was observed on *S. aureus, P. aeruginosa E. coli,* and *E. faecalis,* respectively (Fig. 3A).

**Table 2:** Minimum inhibitory concentration (MIC) of the cLFchimera against some foodborne pathogens

Bacteria	Source	MIC (μg/mL)
*P. aeroginosa*	PAO1	28.6
*S. aureus*	ATCC 15981	93.11
*E. faecalis*	ATCC 47077	3.12
*E. coli*	ATCC 25404	44.33

### Antioxidant activity and thermal stability of recombinant peptide

DPPH as a free radical was used for determining the antioxidant activity of cLFchimera. Our results showed that cLFchimera reacted rapidly with the DPPH. Absorbance at 517 nm was dramatically decreased with increasing the amount of chimeric peptide and reached the minimum at 300 μg/mL. At this peptide concentration (IC50: 300 μg/mL) approximately 50% of DPPH was inactivated by chimeric peptide. Interestingly, by increasing the peptide concentration the antioxidant activity decreased (Fig. 3B). Finally, thermal stability analysis of the chimeric peptide showed that the temperature corresponding to 100 °C for 40 min had no significant effect on its antibacterial activity against *S. aureus* as a Gram-positive bacterial model as shown in Fig. 3C.

### Validation of 3D cLFchimera structure

The generated model for the cLFchimera was examined for overall model quality prior to simulation. The Ramachandran plot obtained for the cLFchimera revealed that 92.3% of the residues were situated within the most favored region, while 7.7% residues of CLFchimera were found within the additional allowed region. This result shows the obtained model was consistent and could be used in molecular dynamics analyses.

### Molecular dynamic analysis

**Fig. 4:**
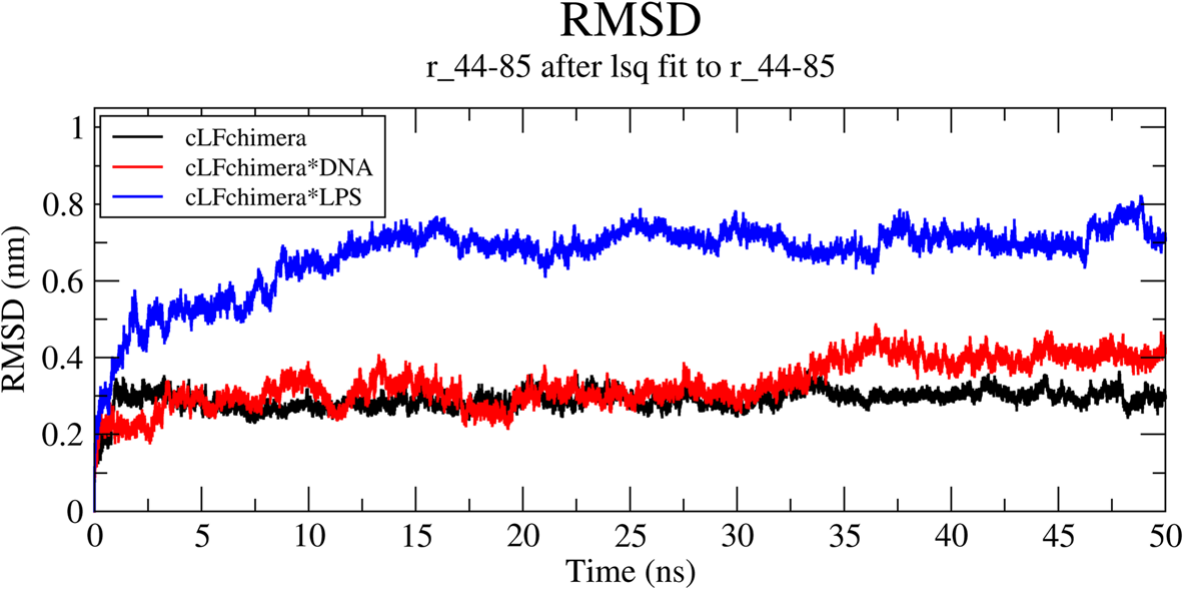
Time evolution of the RMSD, computed through least square fitting of backbone atom

To better understand the possible mode of action for cLFchimera, DNA and LPS were candidates as extra and intra-cellular targets in MD analysis, respectively. RMSD was calculated as one of the most common measures of structural fluctuations during simulation. The results show that interaction of the chimeric peptide to both of the targets increased the RMSD value which indicated the change of peptide structure through binding to these two targets. Change in peptide structure when it interacts with LPS was significantly higher than DNA peptide interaction (Fig. 4).

**Fig. 5:**
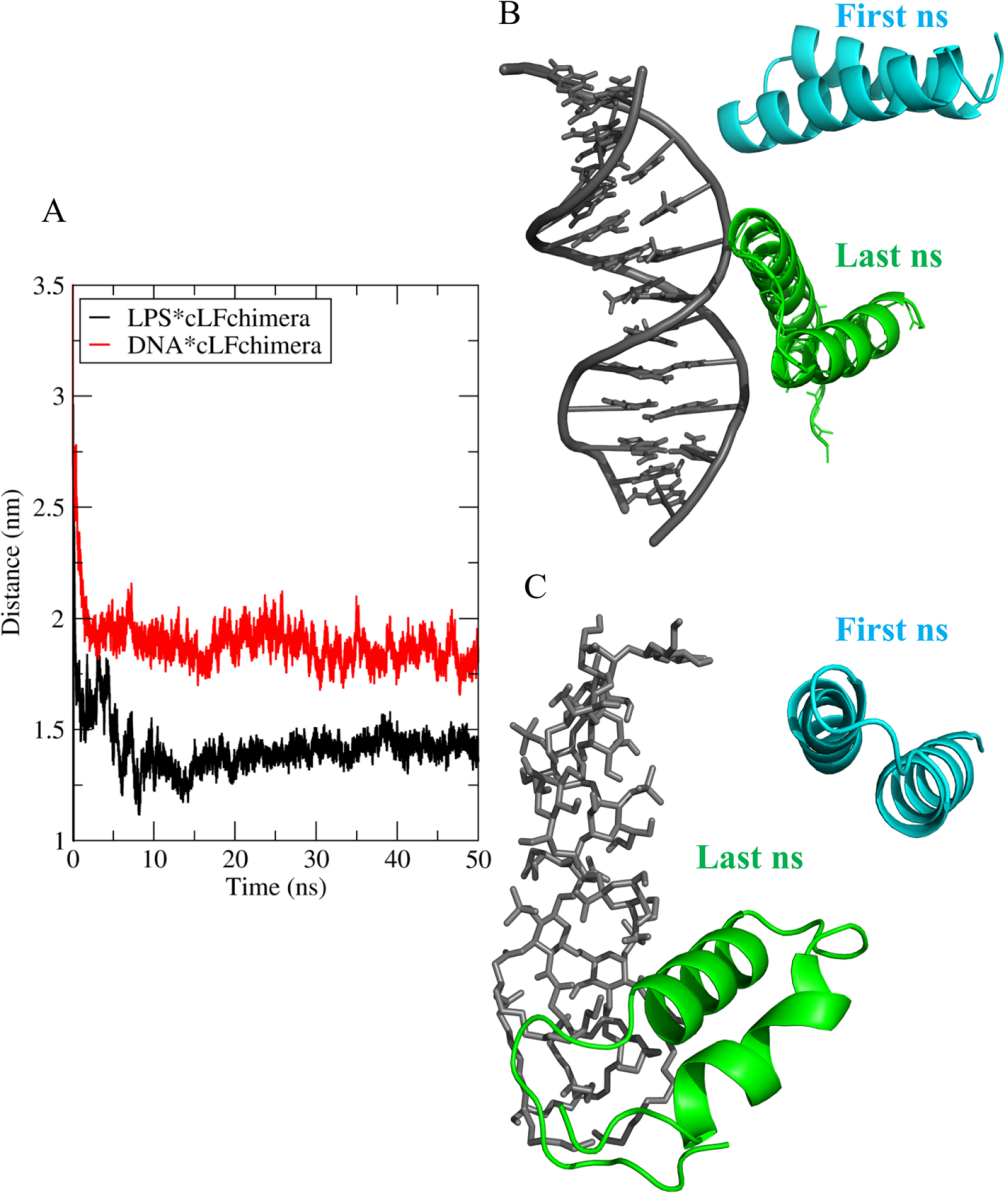
A representative trajectory of the distance decrease during simulation. **a** Center of mass distances for LPS*cLFchimera (red) and DNA*cLFchimera (black). **b** Side view of snapshots at the first and last ns for DNA*cLFchimera interaction. **c** Side view of snapshots at the first and last ns for LPS*cLFchimera interaction

To monitor the distance between peptide and targets during MD simulation the center of mass distance analysis was performed. Side view of snapshots at first and last ns are shown in Fig. 5b and c. COM distances were initially set as approximately 3.5 and 3 nm for cLFchimera*DNA and cLFcimera*LPS, respectively (Fig. 5a and cyan snapshots in Fig. 5b and c). The peptide instantly moved toward the DNA grooves and LPS (Lipid A) and COM distances decreased rapidly compared to their initial state as illustrated in Fig. 5a. and the green snapshots in Fig. 5c. Based on our results the peptide showed high affinity to lipid A as a main target for AMPs in Gram-negative bacteria.

**Fig. 6:**
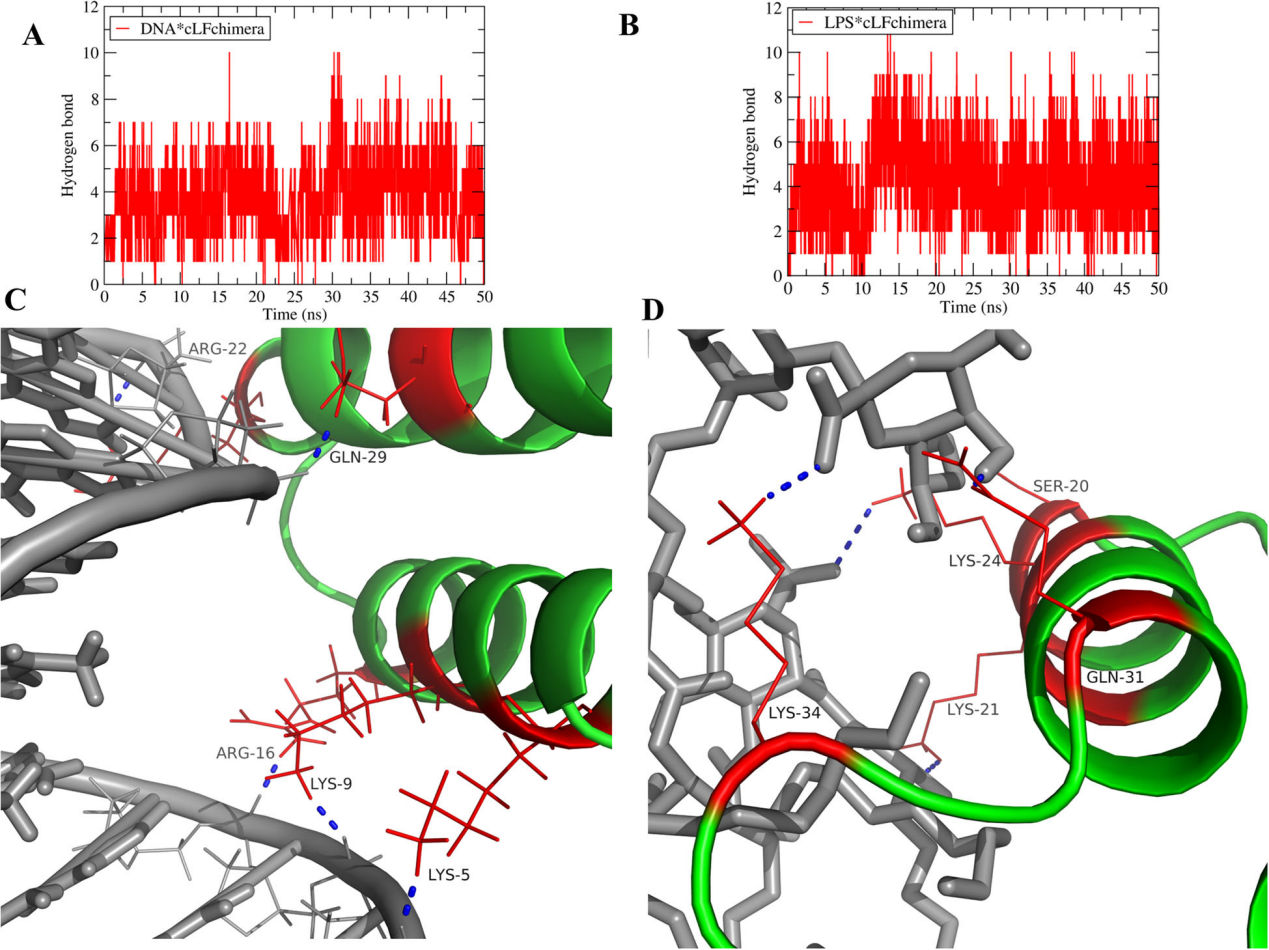
The number of hydrogen bonds between **a** DNA and cLFchimera, **b** LPS and cLFchimera. Snapshot at the t = 50 ns for DNA and cLFchimera (**c**) and LPS and cLFchimera (**d**), the blue dash lines represent the hydrogen bond

For having a deeper view in peptide*LPS and peptide*DNA complexes, the number of hydrogen bonds formed between peptides and targets was calculated (Fig. 6a and b). On average, there were about 5 and 4 hydrogen bonds between the peptide and LPS and DNA, respectively. For example, there were 5 hydrogen bonds in Fig. 6c, between ARG22, GLN29, ARG16, LYS9 and LYS5 and DNA and also 5 hydrogen bonds in Fig. 6d between SER20, LYS24, LYS34, LYS21, and GLN31 and LPS. Considering that hydrogen-bond forming has a main role in stabilizing protein*DNA and protein*LPS complexes, cLFchimera would have a proper stable interaction with these targets. Hydrogen bonding values shown behaved similarly in all replicates.

**Fig. 7:**
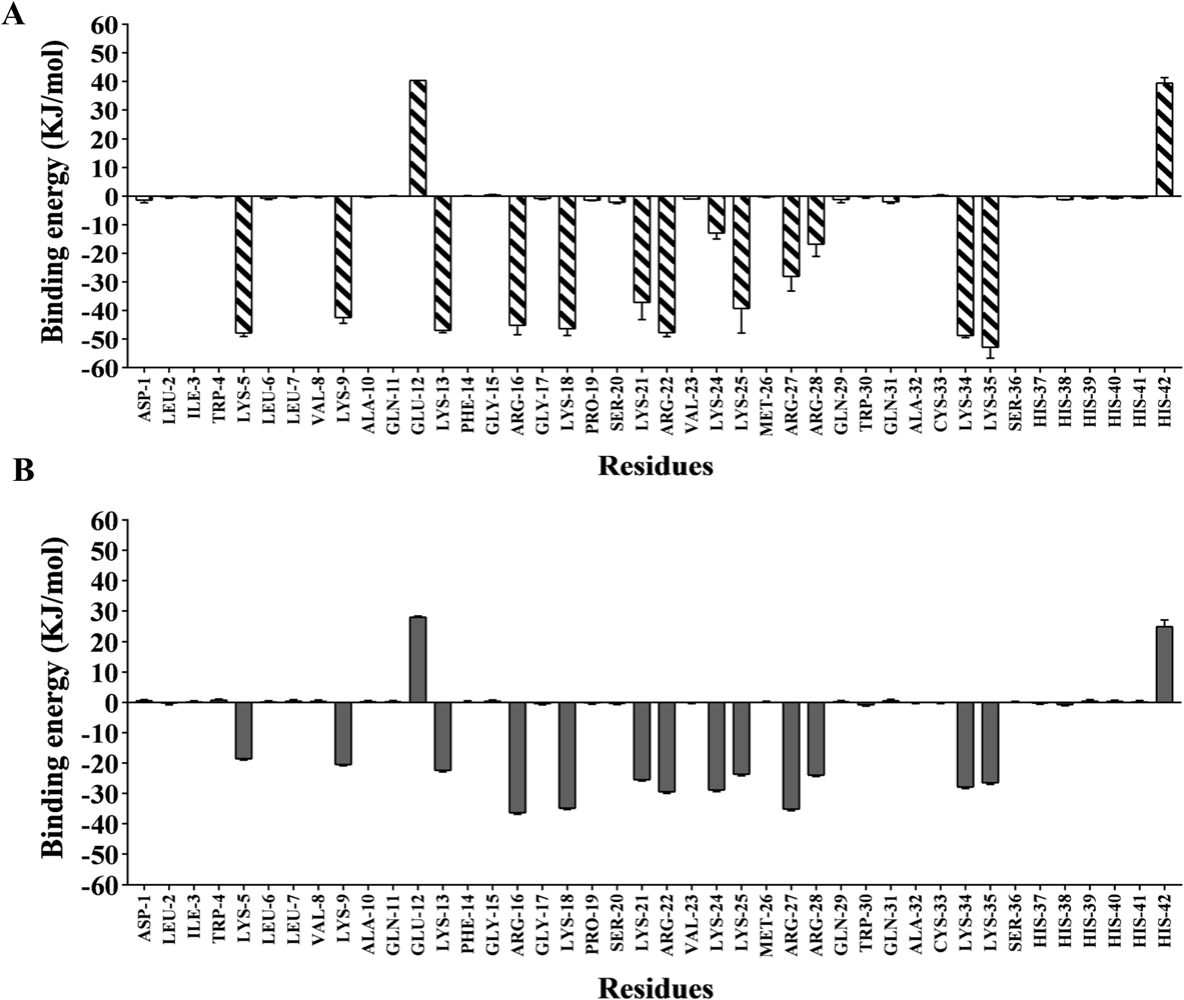
The contribution of residues to the binding energy for active compounds

The binding free energy analysis was calculated using the MM/PBSA method. The results showed that cLFchimera*DNA and cLFchimera*LPS had a strong binding energy 720 kJ/mol and 514 kJ/mol, respectively. The contribution of residues in cLFchimera*DNA and cLFchimera*LPS complexes were screened and the corresponding binding energy was calculated with the MmPbSaDecomp.py python script. The result demonstrated that residues LYS5, LYS9, LYS13, ARG16, LYS18, LYS21, ARG22, LYS24, LYS25, ARG27, ARG28, LYS34 and LYS35 are the key residues for compounds binding in the cLFchimera*DNA and cLFchimera*LPS interactions. However, both GLU12 and SER36 have a negative effect on total binding energy due to their negative charges (Fig. 7).

## Discussion

Generation of engineered food-grade bacteria to produce and deliver therapeutic agents is a hopeful approach for the development of new therapies for gastrointestinal tract disorders [58, 68]. One such bacterium, *L. lactis*, is routinely used for heterologous protein expression in therapeutic and industrial applications [40, 59]. Moreover, *L. lactis* is classified ‘generally recognized as safe’ (GRAS) by the Food and Drug Administration (FDA) [6]. Therefore, this system could be an interesting expression platform in food industry area. As was mentioned above, the main aim of this study was to express an antimicrobial peptide in *L. lactis*. Our results showed that the recombinant camel chimeric lactoferricin and lactoferrampin (cLFchimera) was successfully expressed in *L. lactis* and showed antimicrobial activity against different foodborne pathogens. Our results were well in accordance with other studies which showed antibacterial activity of bovine chimeric lactoferricin and lactoferrampin [8, 69]. (The P170 expression system offers a series of vectors designed for protein secretion by means of SP310mut2, an optimized version of a SP from a native lactococcal protein with the ability to promote efficient protein secretion [29]. The presence of secretion signal peptide SP310mut2 in this expression system allows secretion of recombinant peptide in the medium immediately after synthesis, providing the possibility to use this peptide without any extra process. The efficacy of the P170 expression system for secretion has recently been proven [29] and reconfirmed in our study. The recombinant chimeric peptide was successfully expressed in *L. lactis* strain AMJ1543, a mutant strain which does not have the ability to synthetize Alanine racemase and was previously selected in an alanine-free medium. Due to the lack of antibiotics as a selectable marker, it will be quite safe to use this platform for oral consumption in the future.

The chimeric synthesized recombinant peptide is one of the most hydrophobic peptides. It has been shown that the hydrophobic antimicrobial peptide can recognize the anionic lipids situated in the external surface of bacterial membrane. Attachment of this antimicrobial peptide to the bacterial membrane and subsequently their accumulation inside the membrane causes pores formation and bacterial death [32, 74]. This could be the possible mechanism of antimicrobial activity of the recombinant peptide used in this study.

In the present study we used some foodborne pathogens in the food industry to test the antimicrobial activity of the synthesized recombinant peptide. The desired inhibition was observed against all bacteria treated with the recombinant peptide. Stronger antimicrobial activity was observed in higher peptide concentration. The antibacterial activity of bovine chimeric lactoferricin and lactoferrampin against *Salmonella typhimurium* as a negative and *Staphylococcus aureus* as a Gram-positive bacterium has been proven by other researches and results showed that this recombinant peptide has antibacterial activity against both types of Gram-positive and Gram-negative bacteria [57, 66]. The camel recombinant peptide used in this study also showed an effective antibacterial activity against Gram-positive *S. aureus, E. faecalis* and *L. monocytogenes*, as well as Gram-negative *P. aeruginosa, E. coli* and *S. typhimurium.* Several studies showed that *L. Lactis* has antimicrobial activity against some pathogenic bacteria [3]. Nisin and secreted lactic acid (pH) are two well-characterized antibacterial agents of *L. lactis*. However, in our results we did not observe any inhibitory zone in the disc diffusion assay for culture supernatant of *L. lactis* harboring self-ligated pAMJ1653 vector as negative control after 24 h of incubation even in 40 μL. These observations were likely due to the low concentration of these antibacterial agents in that volume of culture supernatant or short time of incubation.

After purifying the chimeric peptide from culture supernatant, anti-biofilm formation and antioxidant activities were examined. cLFchimera showed strong anti-biofilm formation against *S. aureus* and *P. aeruginosa* and moderate effect on *E. coli* and *E. faecalis*. Wakabayashi et al. [71] showed that both human and bovine lactoferrin had inhibitory effect on biofilm formation against *P. gingivalis* and *P. intermedia* [71]. While anti-biofilm effect of lactoferrin and its derivatives has been demonstrated, the mode of action remains an area of active research [2, 56]. The most obvious mechanism of lactoferrin action is removal of iron in the environment, thus limiting the capacity of the biofilm to survive. However, some evidence suggests that lactoferrin interaction with the biofilm may be more complex [72].

Safaeian and Zabolian [54] reported that oral administration of bovine lactoferrin strongly reduced the production of reactive oxygen species (ROS) by inhibition of oxidative stress [54]. In another study, in vitro antioxidant activity of lactoferrin was evaluated by scavenging capacities against DPPH free radicals and the results showed that lactoferrin had obvious lipid peroxidation inhibitory activity [37]. In parallel with these findings, cLFchimera showed antioxidant activity and reacted rapidly with the DPPH. Interestingly, with increasing the peptide concentration the antioxidant activity decreased which may indicate the peptide interacts with other peptides in high concentration and therefore, loses the opportunity to interact with DPPT as free radical.

The thermal processing method is usually used for different stages of the food industry. For example, it can be important in sterilizing high resistance proteins such as lactoferrin [18]. The thermal stability of lactoferrin has already been reported [13]. The chimeric peptide derived from camel lactoferrin also showed reasonable thermal stability which could be considered in industrial processes.

AMPs usually mediate their antibacterial activity using two well-known mechanisms including extra and intra-cellular actions. LPS [36] and DNA [15] are two candidates considered as the targets of AMPs. Several studies indicated that AMPs initially interact with LPS as the main extra-cellular targets and then mediate their action by cell membrane disruption. The most important parts of LPS for AMPs binding are known as Lipid A [52]. Our in silico analysis showed that cLFchimera had strong affinity to Lipid A which is in accordance with the findings of Rossi et al. [52]. Numerous studies revealed nucleic acids have been proven as intracellular targets for some antimicrobial peptides such as MDpep9 [67], Buforin I [48], Indolicidin [23], Cecropin PR39 [26], and NK18 [73]. Binding of AMPs to major groove of DNA is likely due to positive charge of these peptides which interacts with negative DNA charge [55]. In parallel to this finding, the amount of electrostatic energy binding obtained in our results was the main considerable part of total free energy binding which confirmed the previous results [46]. In both cLFchimera*LPS and cLFchimera*DNA interactions the same residues of peptide acted as active compounds. Based on our contribution analysis, LYS 5, 9, 13, 18, 21, 24, 25, 34, 35 and ARG16, 22, 27 have the negative binding energy, therefore, they are the key residues for these complex formations. According to different charge between peptide and targets these results are rational. However, our results indicate that GLU 12 and HIS 42 were an inhibitor for these interactions. Considering the negative charge of GLU 12 this result was predictable. HIS 42 sometimes has positive charge in physiological pH. So, the presence of this AA as an inhibitor residue in this interaction was a surprising result.

Regarding the possibility of using *L. lactis* expression system in dairy products [25], it seems that our developed expression system in the present study could be used as a food preservative in dairy products. Moreover, the growth ability of *L. lactis* in acidic pH and the optimal stability of this bacterium in the gastrointestinal tract, along with its ability to inhibit pathogenic bacteria in intestine [12] and all other advantages make this bacterium as a suitable candidate in future studies with the aim of its application in the food industry.

## Conclusions

In the present study, we generate a food-grade *L. lactis* strain with the ability to secrete an antimicrobial peptide into the culture medium. The culture supernatant of the engineered *L. lactis* containing the peptide showed significant antibacterial activity against some foodborne pathogens. The purified recombinant peptide had anti-biofilm formation and antioxidant activity and was also stable after boiling. Our in silico analysis showed that cLFchimera had strong affinity to DNA and LPS as two targets in bacteria by positive charged residues after molecular dynamic simulation. Overall, considering the appropriateness and the broad-spectrum antimicrobial activity of this chimeric peptide, it is recommended to evaluate this bioengineered *L. lactis* strain as a food preservative in future studies.

## Data Availability

All data generated or analyzed during this study are included in the article. Data sets from individual experiments can be obtained from the corresponding authors upon reasonable request.

## Abbreviations

AMPs: Antimicrobial peptides
ANOVA: Analysis of variance
CFU: Colony-forming unit
cLFchimera: camel lactoferrin chimera
CLSI: Clinical and Laboratory Standards Institute
COM: Center of mass distances
DPPH: 2,2-Diphenyl-l-picrylhydrazyl
EC_50_: Efficient Concentration
FDA: Food and drug administration
Glc: Glucose
GRAS: Generally recognized as safe
IC_50_: Half maximal inhibitory concentration
LPS: Lipopolysaccharides
MD: Molecular Dynamic
MIC: Minimum inhibitory concentrations
MM/PBSA: Molecular mechanics/Poisson Boltzmann surface area
OD: Optical density
PBS: Phosphate-buffered saline
PCR: Polymerase chain reaction
RMSD: Root mean square deviation
ROS: Reactive oxygen species
SDS-PAGE: SDS polyacrylamide gel electrophoresis

## References

[1] Abraham MJ, Murtola T, Schulz R, Páll S, Smith JC, Hess B, Lindahl E (2015). GROMACS: high performance molecular simulations through multi-level parallelism from laptops to supercomputers. SoftwareX.

[2] Ammons MCB, Ward LS, Fisher ST, Wolcott RD, James GA (2009). in vitro susceptibility of established biofilms composed of a clinical wound isolate of Pseudomonas aeruginosa treated with lactoferrin and xylitol. Int J Antimicrob Agents.

[3] Arqués JL, Rodríguez E, Langa S, Landete JM, Medina M (2015). Antimicrobial activity of lactic acid bacteria in dairy products and gut: effect on pathogens. Biomed Res Int.

[4] Berendsen HJ, Postma JP, van Gunsteren WF, Hermans J (1981). Interaction models for water in relation to protein hydration. Intermolecular forces.

[5] Berendsen HJ, van der Spoel D, van Drunen R (1995). GROMACS: a message-passing parallel molecular dynamics implementation. Comput Phys Commun.

[6] Bermúdez-Humarán LG, Aubry C, Motta JP, Deraison C, Steidler L, Vergnolle N, Chatel JM, Langella P (2013). Engineering lactococci and lactobacilli for human health. Curr Opin Microbiol.

[7] Birollo GA, Reinheimer JA, Vinderola CG (2000). Viability of lactic acid microflora in different types of yoghurt. Food Res Int.

[8] Bolscher JG, Adão R, Nazmi K, van den Keybus PA, van’t Hof W, Amerongen N, Bastos M, Veerman ECI (2009). Bactericidal activity of LFchimera is stronger and less sensitive to ionic strength than its constituent lactoferricin and lactoferrampin peptides. Biochimie.

[9] Bradford MM (1976). A rapid and sensitive method for the quantitation of microgram quantities of protein utilizing the principle of protein-dye binding. Anal Biochem.

[10] Brand-Williams W, Cuvelier ME, Berset CLWT (1995). Use of a free radical method to evaluate antioxidant activity. LWT-Food Sci Technol.

[11] Brogden KA (2005). Antimicrobial peptides: pore formers or metabolic inhibitors in bacteria?. Nat Rev Microbiol.

[12] Coconnier MH, Liévin V, Hemery E, Servin AL (1998). Antagonistic activity against helicobacter infection in vitro and in vivo by the human Lactobacillus acidophilus strain LB. Appl Environ Microbiol.

[13] Conesa C, Calvo M, Sánchez L (2010). Recombinant human lactoferrin: a valuable protein for pharmaceutical products and functional foods. Biotechnol Adv.

[14] Daneshmand A, Kermanshahi H, Sekhavati MH, Javadmanesh A, Ahmadian M (2019). Antimicrobial peptide, cLF36, affects performance and intestinal morphology, microflora, junctional proteins, and immune cells in broilers challenged with E. coli. Sci Rep.

[15] del Castillo FJ, del Castillo I, Moreno F (2001). Construction and characterization of mutations at codon 751 of the Escherichia coli gyrB gene that confer resistance to the antimicrobial peptide microcin B17 and alter the activity of DNA gyrase. J Bacteriol.

[16] Devlieghere F, Vermeulen A, Chitosan DJ (2004). Chitosan: antimicrobial activity, interactions with food components and applicability as a coating on fruit and vegetables. Food Microbiol.

[17] Ebbensgaard A, Mordhorst H, Overgaard MT, Nielsen CG, Aarestrup FM, Hansen EB (2015). Comparative evaluation of the antimicrobial activity of different antimicrobial peptides against a range of pathogenic bacteria. PLoS One.

[18] El-Loly MM, Mahfouz MB (2011). Lactoferrin in relation to biological functions and applications: a review. Int J Dairy Sci.

[19] Eswar N, Webb B, Marti-Renom MA, Madhusudhan MS, Eramian D, Shen MY, Pieper U, Sali A (2006). Comparative protein structure modeling using Modeller. Curr Protoc Bioinformatics.

[20] Evans DJ, Holian BL (1985). The nose–hoover thermostat. J Chem Phys.

[21] Hamzeh-Mivehroud M, Moghaddas-Sani H, Rahbar-Shahrouziasl M, Dastmalchi S (2015). Identifying key interactions stabilizing DOF zinc finger–DNA complexes using in silico approaches. J Theor Biol.

[22] Haney EF, Nazmi K, Bolscher J, Vogel HJ (2012). Structural and biophysical characterization of an antimicrobial peptide chimera comprised of lactoferricin and lactoferrampin. Biochim Biophys Acta.

[23] Hartmann M, Berditsch M, Hawecker J, Ardakani MF, Gerthsen D, Ulrich AS (2010). Damage of the bacterial cell envelope by antimicrobial peptides gramicidin S and PGLa as revealed by transmission and scanning electron microscopy. Antimicrob Agents Chemother.

[24] Helwigh B, Korsgaard H (2007). The community summary report on trends and sources of zoonoses, zoonotic agents, antimicrobial resistance and foodborne outbreaks in the European Union in 2006, European Food Safety Authority.

[25] Hickey CD, Sheehan JJ, Wilkinson MG, Auty MA. Growth and location of bacterial colonies within dairy foods using microscopy techniques: a review. Front Microbiol. 2015;6. 10.3389/fmicb.2015.00099. 10.3389/fmicb.2015.00099 PMC4332360 25741328

[26] Ho YH, Sung TC, Chen CS (2012). Lactoferricin B inhibits the phosphorylation of the two-component system response regulators BasR and CreB. Mol Cell Proteomics.

[27] Holo H, Nes IF (1989). High-frequency transformation, by electroporation, of Lactococcus lactis subsp. cremoris grown with glycine in osmotically stabilized media. Appl. Environ Microbiol.

[28] Hou ZJL, Fang C, Zhou Y, Bai H, Zhang X, Luo X (2011). Underlying mechanism of in vivo and in vitro activity of C-terminal-amidated Thanatin against clinical isolates of extended-spectrum β-lactamase–producing Escherichia coli. J Infect Dis.

[29] Jørgensen CM, Vrang A, Madsen SM (2014). Recombinant protein expression in Lactococcus lactis using the P170 expression system. FEMS Microbiol Lett.

[30] Klingenberg C, Aarag E, Ronnestad A, Sollid JE, Abrahamsen TG, Kjeldsen G (2005). Coagulase-negative staphylococcal sepsis in neonates. Association between antibiotic resistance, biofilm formation and the host inflammatory response. Pediatr Infect Dis J.

[31] Kollman PA, Massova I, Reyes C, Kuhn B, Huo S, Chong L, Lee M, Lee T, Duan Y, Wang W, Donini O (2000). Calculating structures and free energies of complex molecules: combining molecular mechanics and continuum models. Acc Chem Res.

[32] Kouzayaha, Nasir M, Buchet R (2009). Antimicrobial peptides and their use in medicine Phys. Chem B.

[33] Kumari R, Kumar R, Open Source Drug Discovery Consortium, Lynn A. g_mmpbsa—A GROMACS tool for high-throughput MM-PBSA calculations. J Chem Inf Model. 2014;54(7):1951–62. 10.1021/ci500020m 24850022

[34] Laskowski RA, MacArthur MW, Moss DS, Thornton JM (1993). PROCHECK: a program to check the stereochemical quality of protein structures. J Appl Crystallogr.

[35] Lemes A, Sala L, Ores J, Braga A, Egea M, Fernandes K (2016). A review of the latest advances in encrypted bioactive peptides from protein-rich waste. Int J Mol Sci.

[36] Leon-Sicairos N, Canizalez-Roman A, de la Garza M, Reyes-Lopez M, Zazueta-Beltran J, Nazmi K, Gomez-Gil B, Bolscher JG (2009). Bactericidal effect of lactoferrin and lactoferrin chimera against halophilic Vibrio parahaemolyticus. Biochimie.

[37] Li T, Wang C, Yan X (2012). Antioxidant activity of Lactoferrin in vitro. Food Sci.

[38] Linde A, Ross CR, Davis EG, Dib L, Blecha F, Melgarejo T (2008). Innate immunity and host defense peptides in veterinary medicine. J Vet Intern Med.

[39] Mason CK, Collins MA, Thompson K (2005). Modified electroporation protocol for lactobacilli isolated from the chicken crop facilitates transformation and the use of a genetic tool. J Microbiol Methods.

[40] McKay LL, Baldwin KA (1990). Applications for biotechnology: present and future improvements in lactic acid bacteria. FEMS Microbiol Lett.

[41] Norrby K, Mattsby-Baltzer I, Innocenti M, Orally TS (2001). Bovine lactoferrin systemically inhibits VEGF165-mediated angiogenesis in the rat. Int J Cancer.

[42] Nosé S, Klein ML (1983). Constant pressure molecular dynamics for molecular systems. Mol Phys.

[43] Oliver SP, Jayarao BM, Almeida RA (2005). Foodborne pathogens in milk and the dairy farm environment: food safety and public health implications. Foodbourne Pathog Dis.

[44] Omwandho CO, Kubota T (2010). Salmonella enterica serovar Enteritidis: a mini-review of contamination routes and limitations to effective control. Jpn Agric Res Q.

[45] Oostenbrink C, Villa A, Mark AE, Van Gunsteren WF (2004). A biomolecular force field based on the free enthalpy of hydration and solvation: the GROMOS force-field parameter sets 53A5 and 53A6. J Comput Chem.

[46] Pandey B, Grover A, Sharma P (2018). Molecular dynamics simulations revealed structural differences among WRKY domain-DNA interaction in barley (Hordeum vulgare). BMC Genomics.

[47] Parada JL, Caron CR, Medeiros ABP, Soccol CR (2007). Bacteriocins from lactic acid bacteria: purification, properties and use as biopreservatives. Braz Arch Biol Technol.

[48] Park CB, Kim HS, Kim SC (1998). Mechanism of action of the antimicrobial peptide buforin II: buforin II kills microorganisms by penetrating the cell membrane and inhibiting cellular functions. Biochem Biophys Res Commun.

[49] Pirkhezranian Z, Tanhaeian A, Mirzaii M, Sekhavati MH. Expression of Enterocin-P in HEK platform: evaluation of its cytotoxic effects on Cancer cell lines and its potency to interact with cell-surface glycosaminoglycan by molecular modeling. Int J Pept Res Ther. 2019;19(4):1–10.

[50] Pirkhezranian Z, Tahmoorespur M, Monhemi H, Sekhavati MH. Computational peptide engineering approach for selection the best engendered camel Lactoferrin-derive peptide with potency to interact with DNA. Int J Pept Res Ther. 2020a;19(6):1–10.

[51] Pirkhezranian Z, Tahmoorespur M, Daura X, Monhemi H, Sekhavati MH (2020). Interaction of camel Lactoferrin derived peptides with DNA: a molecular dynamics study. BMC Genomics.

[52] Rossi P, Giansanti F, Boffi A, Ajello M, Valenti P, Chiancone E, Antonini G (2002). Ca2+ binding to bovine lactoferrin enhances protein stability and influences the release of bacterial lipopolysaccharide. Biochem Cell Biol.

[53] Roy M, Kuwabara Y, Hara K, Watanabe Y, Tamai Y (2002). Peptides from the N-terminal end of bovine lactoferrin induce apoptosis in human leukemic (HL-60) cells. J Dairy Sci.

[54] Safaeian L, Zabolian H. Antioxidant effects of bovine lactoferrin on dexamethasone-induced hypertension in rat. ISRN pharmacology, 2014. 10.1155/2014/943523 PMC3920649 24587916

[55] Sim S, Wang P, Beyer BN, Cutrona KJ, Radhakrishnan ML, Elmore DE (2017). Investigating the nucleic acid interactions of histone-derived antimicrobial peptides. FEBS Lett.

[56] Singh PK, Parsek MR, Greenberg EP, Welsh MJ (2002). A component of innate immunity prevents bacterial biofilm development. Nature.

[57] Sinha M, Kaushik S, Kaur P, Sharma S, Singh TP. Antimicrobial lactoferrin peptides: the hidden players in the protective function of a multifunctional protein. Int J Pept. 2013;2013. 10.1155/2013/390230. 10.1155/2013/390230 PMC3608178 23554820

[58] Steidler L (2003). Genetically engineered probiotics. Best Pract Res Clin Gastroenterol.

[59] Steidler L, Hans W, Schotte L, Neirynck S, Obermeier F, Falk W, Fiers W, Remaut E (2000). Treatment of murine colitis by *Lactococcus lactis* secreting interleukin-10. Science.

[60] Stepanović S, Vuković D, Hola V (2007). Quantification of biofilm in microtiter plates:overview of testing conditions and practical recommendations for assessment ofbiofilm production by staphylococci. APMIS.

[61] Tahmoorespur M, Azghandi M, Javadmanesh A, Sekhavati MZ, MH. A novel chimeric anti-HCV peptide derived from camel Lactoferrin and molecular level insight on its interaction with E2. Int J Pept Res Ther. 2019;10(4):1–13.

[62] Tanhaeian A, Ahmadi FS, Sekhavati MH, Mamarabadi M (2018). Expression and purification of the main component contained in camel milk and its antimicrobial activities against bacterial plant pathogens. Probiotics Antimicrob Proteins.

[63] Tanhaeian A, Jaafari MR, Ahmadi FS, Vakili-Ghartavol R, Sekhavati MH. Secretory expression of a chimeric peptide in Lactococcus lactis: assessment of its cytotoxic activity and a deep view on its interaction with cell-surface Glycosaminoglycans by molecular modeling. Probiotics Antimicrob Proteins. 2018d;11(3):1–8. 10.1007/s12602-018-9496-6 30552573

[64] Tanhaiean A, Azghandi M, Razmyar J, Mohammadi E, Sekhavati MH. Recombinant production of a chimeric antimicrobial peptide in E. coli and assessment of its activity against some avian clinically isolated pathogens. Microb Pathog. 2018a;122:73–8. 10.1016/j.micpath.2018.06.012 29890331

[65] Tanhaieian A, Sekhavati MH, Ahmadi FS, Mamarabadi M (2018). Heterologous expression of a broad-spectrum chimeric antimicrobial peptide in Lactococcus lactis: its safety and molecular modeling evaluation. Microb Pathog.

[66] Tang XS, Tang ZR, Wang SP, Feng ZM, Zhou D, Li TJ, Yin YL (2012). Expression, purification, and antibacterial activity of bovine lactoferrampin–lactoferricin in *Pichia pastoris*. Appl Biochem Biotechnol.

[67] Tang YL, Shi YH, Zhao W, Hao G, Le GW (2009). Interaction of MDpep9, a novel antimicrobial peptide from Chinese traditional edible larvae of housefly, with Escherichia coli genomic DNA. Food Chem.

[68] Van de Guchte M, Kok J, Venema G (1992). Gene expression in Lactococcus lactis. FEMS Microbiol Lett.

[69] Van der Kraan MI, Groenink J, Nazmi K, Veerman EC, Bolscher JG, Amerongen AVN (2004). Lactoferrampin: a novel antimicrobial peptide in the N1-domain of bovine lactoferrin. Peptides.

[70] Van Der Spoel D, Lindahl E, Hess B, Groenhof G, Mark AE, Berendsen HJ (2005). GROMACS: fast, flexible, and free. J Comput Chem.

[71] Wakabayashi H, Yamauchi K, Kobayashi T, Yaeshima T, Iwatsuki K, Yoshie H (2009). Inhibitory effects of lactoferrin on growth and biofilm formation of Porphyromonas gingivalis and Prevotella intermedia. Antimicrob Agents Chemother.

[72] Xiao R, Kisaalita WS (1997). Iron acquisition from transferrin and lactoferrin by Pseudomonas aeruginosa pyoverdin. Microbiology.

[73] Yan J, Wang K, Dang W, Chen R, Xie J, Zhang B, Song J, Wang R (2013). Two hits are better than one: membrane-active and DNA binding-related double-action mechanism of NK-18, a novel antimicrobial peptide derived from mammalian NK-lysin. Antimicrob Agents Chemother.

[74] Yang L, Harroun TA, Weiss TM, Ding L, Huang HW (2001). Barrel-stave model or toroidal model? A case study on melittin pores. Biophys J.

